# Effectiveness of remote ischemic preconditioning in patients undergoing transplant surgery: meta-analysis of randomized control studies

**DOI:** 10.1097/MS9.0000000000002306

**Published:** 2024-07-05

**Authors:** Ameer Fadhel Abbas, Haania Shahbaz, Armand Gumera, Ali Saad Al-Shammari, Mohanad Mahdey Salih Alchamaley, Hashim Talib Hashim, Mohannad Abdeltawwab, Mahmoud Amin

**Affiliations:** a Department of surgery, University of Al-Qadisiyah College of Medicine, Al Diwaniyah; b Imam Ali General Hospital, Baghdad; c University of Warith Al-Anbiyaa, College of Medicine, Karbala, Iraq; d Dow University of Health Sciences, Karachi, Pakistan; e Department of Surgery, University of Melbourne, Melbourne, VIC, Australia; f Faculty of Medicine, Fayoum University, Fayoum, Egypt

**Keywords:** kidney transplant, organ transplantation, remote ischemic preconditioning, RIC, RIPC, transplant

## Abstract

**Introduction:**

Remote ischemic preconditioning (RIPC) is a phenomenon in which the induction of shortened periods of ischemia prior to surgical procedures within a distant tissue preserves other tissues or organs of concern, such as the liver or kidney in transplant surgery, in the event of prolonged ischemic insults. The authors aim to evaluate the effectiveness of RIPC in patients undergoing transplant surgery, specifically kidney and liver transplants.

**Materials and methods:**

PubMed, Embase, and Scopus were searched until 19 December 2023 for trials evaluating RIPC in patients undergoing transplant surgery. A total of 9364 search articles were obtained, which yielded 10 eligible studies. Data analysis was done using RevMan 5.4 software. The risk of bias was done using Cochrane risk of bias tool.

**Results and discussion:**

For graft rejection, the study observed a relative risk of 0.99 (95% CI, 0.49–1.98, *P*=0.97) from 5 trials, indicating no significant effect of RIPC on graft survival in both kidney and liver transplants. The length of hospital stay also showed no significant decrease for those undergoing RIPC, with mean difference (MD) of -0.58 (95% CI, −1.38 to 0.23, *P*=0.16). GFR at 1-year post-kidney transplant did not significantly change in the RIPC group compared to controls, as evidenced by an MD of -0.13 (95% CI, −3.79 to 3.54, *P*=0.95). These results collectively suggest that RIPC may not be effective in reducing patient, or graft, outcomes.

## Introduction

Highlights

- This review examined the effectiveness of remote ischemic preconditioning (RIPC) in liver and kidney transplant surgeries; data from 10 randomized trials were assessed.
- Our analysis showed no significant impact on graft survival, mortality, or hospital stay.
- While RIPC showed short-term benefits like improved early kidney function, long-term outcomes remained unaffected.
- Studies on RIPC focused on recipients, with unclear benefits if preconditioning occurred in donors.
- Studies suggest potential harm in RIPC grafts due to increased injury markers. Unlike in myocardial infarction, RIPC didn’t reduce mortality in transplant cases.
- Ischemic reperfusion injury (IRI) contributes to organ damage post-transplant, prompting exploration of methods like RIPC to mitigate it, yet results are inconclusive.
- Propofol, an anesthetic, may inhibit RIPC, raising questions about its efficacy.
- Further research is needed, especially concerning low-quality grafts and the long-term effects of RIPC.

Remote ischemic preconditioning (RIPC) is a phenomenon in which the induction of shortened periods of ischemia prior to surgical procedures (preconditioning) within a distant tissue (remote ischemia) preserves other tissues or organs of concern, such as the liver or kidney in transplant surgery, in the event of prolonged ischemic insults^1^. The typical procedural protocol is performed through the inflation of a blood pressure cuff on distant body limbs in short cycles so as to cause transient limb ischemia^2^. Ischemic preconditioning was first described in 1986, when findings supported the protective role of RIPC on the myocardium of a canine model^3^. There have been many hypotheses for the mechanism of RIPC^4^, but it is still not fully understood. One hypothesis is that remote ischemic preconditioning activates the humoral and neural pathways by opening the mitochondrial ATP-sensitive myocardial potassium channels and closing mitochondrial permeability transition pores, consequently decreasing the cardiomyocytes vulnerability to ischemia-induced cell death^5^. The incidence of complications after organ transplantation is high, frequently leading to a series of clinical sequelae that impair patient recovery and prognosis^6^. As a result, there is a need for a systematic review to evaluate this intervention. However, while human and animal studies have suggested potential benefits of RIPC in organ transplant patients, the current state of research remains inconclusive^7,8^. The body of evidence presents a variable degree of quality, and no consensus has been reached regarding the efficacy of RIPC in organ transplant patients. The aim of this systematic review is to evaluate the effectiveness of remote ischemic preconditioning in patients undergoing transplant surgery, specifically kidney and liver transplants. By abiding by proper methodology, this systematic review can offer insights that have the potential to support decision-making in the management of patients undergoing organ transplantation.

## Methods

### Literature search

We adhered to guidelines established by the Preferred Reporting Items for Systematic Review and Meta-Analysis (PRISMA, Supplemental Digital Content 1, http://links.lww.com/MS9/A550) for our systematic review and meta-analysis^9^. We registered our protocol with the International PROSPERO Registry for Systematic Reviews and Meta-Analyses with ID number CRD42023494447. The results from included studies are all publicly available.

### Data sources and search strategy

Comprehensive searches were conducted across various databases, including PubMed, EMBASE and Scopus. The search encompassed articles from the inception of records until 19 December 2023. A meticulous approach, employing specific keywords and Medical Subject Headings (MeSH) terms that are: (remote ischemic precondition* OR RIPC* OR remote ischemic condition*) AND (Transplant OR Graft* OR Grafting* OR Transplantation*).

Additionally, manual screening of references from retrieved trials, prior meta-analyses, and review articles, along with an examination of citations from relevant articles on Google Scholar, was performed to ensure a comprehensive review of available literature. Attempts were made to contact authors for data through e-mail where applicable, although responses were not obtained.

### Eligibility criteria and outcomes

The study selection criteria followed the PICO (Population, Intervention, Comparison, and Outcomes) framework, encompassing specific parameters: (i) involving organ transplant recipients, (ii) assessing the impact of remote ischemic preconditioning as an intervention, (iii) compared to control groups without this intervention, and (iv) examining various outcomes related to graft survival, length of hospital stay, and organ functioning at follow-up. We hypothesized that RIPC would lead to reduced graft rejection rates. Exclusion criteria involved studies focusing on non-transplantation settings or those focusing on ischemic preconditioning instead of remote ischemic preconditioning. Furthermore, case reports, articles not in English, review articles, and abstracts lacking full-text availability were excluded from consideration.

### Study selection and data extraction

Two independent reviewers systematically screened titles and abstracts to identify studies that met the predefined eligibility criteria. Any discrepancies in study selection were resolved through consensus between the reviewers. Selected studies underwent a thorough full-text review to extract relevant data. Key information including author details, study design, patient demographics, baseline characteristics, and reported outcomes were extracted using a pre-designed Microsoft Excel sheet. The outcomes for rejection rates of grafts, length of hospital stays, and functioning of kidneys and livers were extracted, and only studies with data available for certain time-points were included. Attempts were made to fill in missing data from graphical data in studies included.

### Quality assessment of included studies

The quality assessment of the included studies was conducted using the randomized controlled trial (RCT) Bias Risk Assessment Tool^10^, which is recommended for randomized controlled trials in Cochrane Handbook 5.1. This assessment evaluated the selection criteria of studies, comparability between groups, exposure, and outcomes. Two researchers independently performed the assessment, and any discrepancies were resolved through discussion and consensus. The results of the evaluation are present in the supplemental digital content file (SDC, Supplemental Digital Content 2, http://links.lww.com/MS9/A551).

### Data synthesis and heterogeneity

Data analysis was carried out using RevMan, Version 5.4, employing the Mantel-Haenszel random-effects model. Results were presented as risk ratios (RR) with corresponding 95% CIs for dichotomous outcomes. The Higgins I^2^ test was utilized to assess heterogeneity among studies, categorizing it as low, moderate, or high. A leave-one-out method was done for sensitivity analysis to determine the robustness of the results. Subgroup analysis was done to reduce heterogeneity, particularly for outcomes involving different organs. The forest plots were generated as graphics from the software.

### Literature search

**Figure 1:**
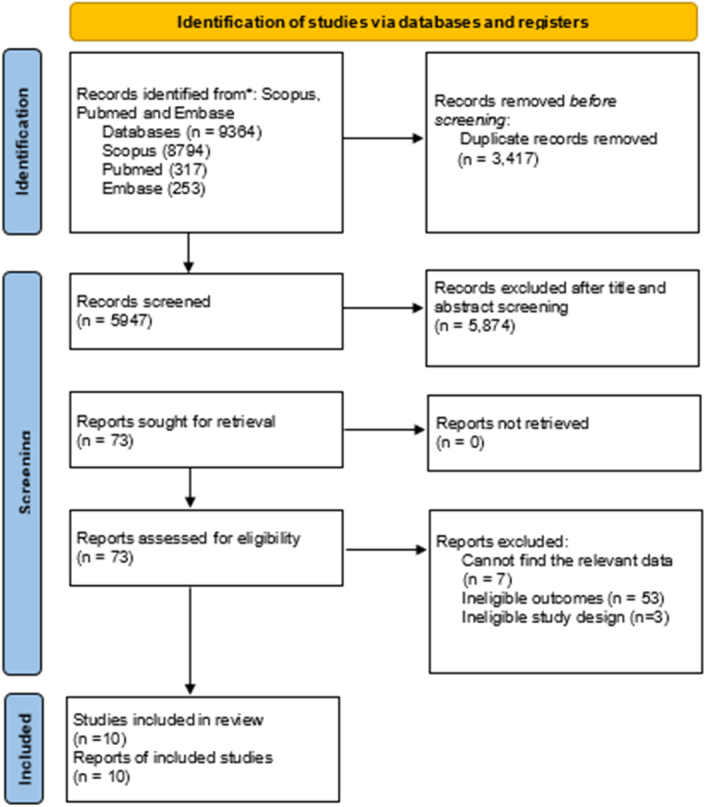
Flow chart of study selection and search result based on Preferred Reporting Items for Systematic Review and Meta-Analysis (PRISMA) 2020.

The initial literature search turned up 9364 articles in all. Articles were screened for title and abstract after duplicates were removed using Rayyan, and 73 of those were found to be eligible for full-text screening. The 10 articles for this systematic review and meta-analysis were chosen after thorough and meticulous full-text screening. The full process is highlighted in Figure 1.

## Results

**Figure 2:**
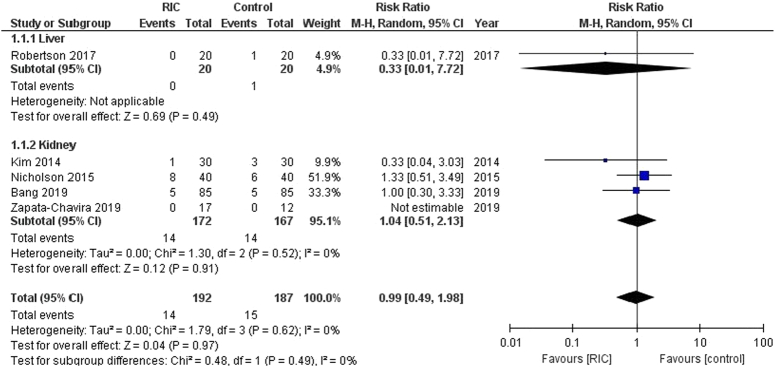
Forest plot of comparison: RIPC vs. control, outcome: graft rejection. RIC, remote ischemic preconditioning.

**Figure 3:**
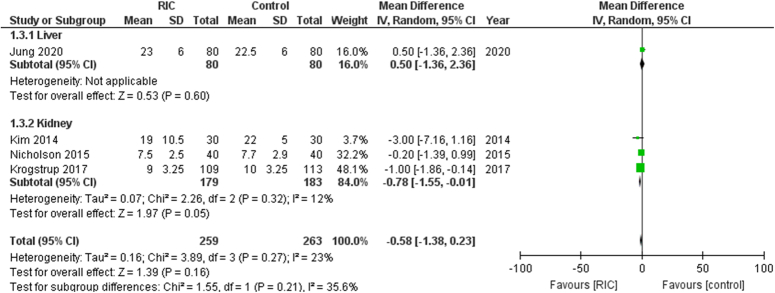
Forest plot of comparison: RIPC vs. control, outcome: length of hospital stay. RIC, remote ischemic preconditioning.

In 10 randomized controlled trials, with 604 participants in the RIPC and 603 in the control group, a total of 1207 participants, divided based on kidney organ transplant or liver organ transplant, RIPC was compared with control methods (placebo, sham surgery). The baseline characteristics of the population include age, sex, diabetes, hypertension, preoperative glomerular filtration rate (GFR) and serum creatinine for patients receiving kidney transplant, and age, gender, Model for End-Stage Liver Disease (MELD) score, aspartate aminotransferase (AST), and alanine transaminase (ALT) in the liver organ transplant patients (SDC; table 1, Supplemental Digital Content 2, http://links.lww.com/MS9/A551, table 2). Most studies were at low risk of bias (SDC; Fig. 3). Additional outcomes have also been presented (SDC; Figs. 1 and 2, Supplemental Digital Content 2, http://links.lww.com/MS9/A551).

### Primary outcome

#### Graft rejection

In this analysis involving 379 patients from five trials, RIPC methods were associated with neither a significant increase nor decrease in the risk of graft rejection compared to control group. [RR: 0.99 (95% CI, 0.49–1.98, *P*=0.97), Fig. 2]. There was no observed heterogeneity among the trials (I²=0%). For the subgroups, the liver graft rejection analysis involved one trial [RR: 0.33 (95% CI, 0.01–7.72, *P*=0.49)], and the kidney graft rejection analysis involved four trials [RR: 1.04 (95% CI, 0.51–2.13, *P*=0.91)]. Both subgroups showed no significant heterogeneity.

### Secondary outcomes

#### Length of hospital stay

In this analysis comprising 522 patients across 4 studies, the length of hospital stay did not significantly decrease in patients receiving RIPC (Fig. 3). The mean difference (MD) was −0.58 [95% CI, −1.38 to 0.23, *P*=0.16]. The heterogeneity among the included studies was low (I²=23%).

The liver subgroup had one trial [MD: 0.50 (95% CI, −1.36 to 2.36, *P*=0.60)]. The kidney subgroup had 3 trials [MD: −0.78 (95% CI, −1.55 to −0.01, *P*=0.05)].

### GFR at 1 year in kidney transplant patients

**Figure 4:**
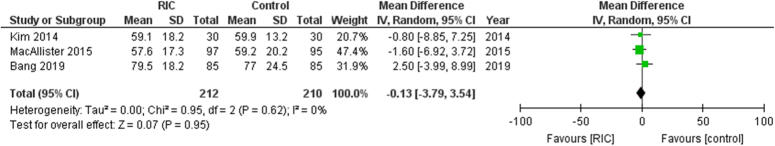
Forest plot of comparison: RIPC vs. control, outcome: glomerular filtration rate at 1 year. RIC, remote ischemic preconditioning.

There were 422 patients evaluated and the analysis showed that RIPC did not significantly change GFR at 1-year post-transplant when compared with the controls (Fig. 4). The aggregated MD was −0.13 [95% CI, −3.79 to 3.54, *P*=0.95], suggesting no statistical significance of the intervention. Additionally, the heterogeneity of the included studies was non-existent (I²=0%), indicating consistency across the study outcomes.

## Discussion

Our review evaluated the efficacy of RIPC in the context of transplant surgery, focusing on liver and kidney transplants, analyzing three main outcomes: graft rejection, GFR at 1 year, length of hospital stay. Additional outcomes are present in the online resource. The data from 10 randomized trials offer insights into its effectiveness. Results do not show a statistically significant impact of RIPC on graft survival. Likewise, mortality at follow-up (online resource) remained the same for both groups as did the length of hospital stay, suggesting no overall improvement in the clinical outcomes as a result of the procedure.

Ischemic reperfusion injury (IRI), which occurs due to revascularization of the transplanted organ after a period of ischemia following removal from a donor, particularly a deceased one, leads to organ damage and delayed graft function, which ultimately affects the survival of the graft^11^.

Several studies have sought to find means of reducing ischemic reperfusion injury in transplanted grafts, and ischemic preconditioning, both remote and to the target organ, has been one of the theories, which, however, has produced mixed results^12^. With RIPC, graft loss rates have been reported to be similar in both RIPC and non-RIPC groups by several studies^13^. Since most studies focused on RIPC on recipients, the benefit of such an intervention to be improved had the preconditioning has been done in the donor is not clear, a feat that is, nonetheless, extremely hard to achieve because of the organ being donated after death in some cases. Additionally, no survival benefit was seen in the transplant recipient, as corroborated by other studies^13,14^. However, Propofol, seen to inhibit RIPC, was used in several of these studies as observed by Veighey^13^. The benefit of RIPC unhindered by anesthetic agents remains to be studied.

From a clinical perspective, though these findings do not conclusively support the effectiveness of RIPC, they do suggest trends that may warrant further research. While a previous meta-analysis demonstrated the improved benefits of ischemic preconditioning (IPC) of the organ to be transplanted on mortality and AST levels, it failed to factor in the benefits of RIPC, suggesting that IPC could result in better outcomes despite the feasibility of the latter^15^.

Kim *et al.* ^16^ reported a faster reduction of serum creatinine and an increased urine output in the first 24 h, but the superiority of RIPC was limited to this time, following which the GFR levels were similar in both groups, and this limited post-operative benefit were further corroborated by other studies^17,18^. However, long-term outcomes like graft dysfunction and failure tended to be unaffected. What impact RIPC would have on ‘low-quality’ grafts prone to delayed graft function was not reported, and this gap in literature might well be answered by future trials. In fact, Nicholson *et al.* ^14^ suggested increased ischemic injury to the RIPC grafts due to increased levels of kidney injury markers when compared to the control group. Since studies have reported decreased mortality with myocardial infarction following RIPC, it is not certain what causes a difference in cases of transplant^19^. This has held for liver transplant studies as well^20^.

### Limitations

There were significant challenges in this review to report on parameters such as ischemic markers and any changes through RIPC. This is due to the lack of adequate data in the included studies, restricting our ability to draw conclusions on the impact of RIPC post-transplant. Furthermore, many of the included studies have relatively small sample sizes, raising concerns regarding the generalizability and statistical power of our findings. There is also methodological variability used for ischemic preconditioning. Some studies administered anesthesia prior to the preconditioning procedure, while others did so after, and the techniques for inducing ischemic preconditioning varied. These differences have the potential to influence outcomes, which can make it challenging to compare results across studies. Finally, our review fails to account for differences in graft quality across studies. Graft quality is an important factor of post-transplant outcomes. Without a comprehensive evaluation of graft quality and its impact on RIPC efficacy, our understanding of the true effectiveness of this intervention remains unfinished.

## Conclusions

The findings of this review do not offer definitive evidence to support the routine use of RIPC in transplant surgery. Future research should consider investigating the effects of RIPC in specific subpopulations of transplant patients or under different clinical conditions where preconditioning may demonstrate effectiveness.

## Ethical approval

No informed consent or institutional permission was required for the review.

## Consent

Informed consent was not required for this systematic review.

## Source of funding

The authors declare that they have no financial interests or affiliations that could influence the results or interpretation of this systematic review.

## Author contribution

All authors have equal contributions.

## Conflicts of interest disclosure

The authors involved in this review certify that they have no conflict of interest, affiliations with or involvement in any organization or entity with any financial interest.

## Research registration unique identifying number (UIN)

CRD42023494447.

## Guarantor

Ameer Fadhel Abbas.

## Data availability statement

Not applicable.

## Provenance and peer review

Not applicable.

## Supplementary Material
