# Supplementary material for: Effectiveness of remote ischemic preconditioning in patients undergoing transplant surgery: meta-analysis of randomized control studies
Source: Ann Med Surg (Lond). 2024 Jul 5;86(9):5455–60. doi: 10.1097/MS9.0000000000002306 (PMC11374220; doi:10.1097/MS9.0000000000002306)
Supplement: Supplementary file 2 [file ms9-86-5455-s002.doc]

**Authors:**

Ameer Fadhel Abbas^1^**,** Haania Shahbaz^2^, Armand Gumera^3^, Ali Saad Al-Shammari^4^, Mohanad Mahdey Salih Alchamaley^1^, Hashim Talib Hashim^5^, Mohannad Abdeltawwab^6^, Mahmoud Amin^6^.

1. Ameer Fadhel Abbas
2. Haania Shahbaz

ORCID ID: 0000-0002-7343-6353

1. Armand Gumera

ORCID ID: 0000-0003-3780-0514

1. Ali Saad Al-Shammari

ORCID ID: 0000-0002-4298-5456

1. Mohanad Mahdey Salih Alchamaley

ORCID ID: 0009-0004-1933-4139

1. Hashim Talib Hashim
2. Mohannad Abdeltawwab (Corresponding author)

mj1207@fayoum.edu.eg

ORCID ID: 0009-0001-3307-8600

1. Mahmoud Amin

**Author Affiliations**

1. Department of surgery, University of Al-Qadisiyah College of Medicine, Iraq
2. Dow University of Health Sciences, Karachi, Pakistan
3. Department of Surgery, University of Melbourne, Melbourne, Australia
4. Imam Ali General Hospital, Baghdad, Iraq
5. University of Warith Al-Anbiyaa, College of Medicine, Karbala, Iraq
6. Faculty of Medicine - Fayoum University, Fayoum, Egypt

|  | | | | | | | | |
| --- | --- | --- | --- | --- | --- | --- | --- | --- |
| Study name | Study type | Study Population | Age | Males | Diabetes | Hypertension | Serum Cr mg/dl | GFR mL/min/1.73 m^2^ |
| Bang 2019 [9] | RCT | 340 | RIC 47.6 ± 10.0  Control 45.9 ± 13.4 | RIC 54.1%  Control:Control 50.6 % | RIC 25.9%  Control 21.2% | RIC 82.4%  Control 89.4% | RIC 7.5 (6.0–9.1)  Control 7.9 (5.7–9.2) | RIC 7.0 (5.0–9.0)  Control 7.0 (5.0–9.0) |
| Krogstrup 2017 [10] | RCT | 220 | RIC 58.1 (49.5–65.0)  Control 61.4 (49.4–66.6) | RIC 60%  Control 61% | RIC 17%  Control 22% | RIC 88%  Control 92% | NR | NR |
| Nicholson 2015 [11] | RCT | 80 | RIC 45 ± 14  Control 47 ± 14 | RIC 67.5%  Control 52.5% | RIC 10%  Control 0% | RIC 30% Control 20% | NR | NR |
| Kim 2014  [12] | RCT | 60 | RIC 49 (39-52)  Control 46 (36-50) | RIC 66.6%  Control 70% | RIC 27%  Control 27% | RIC 27%  Control 33% | RIC 8.86 (3.10)  Control 8.70 (4.01) | NR |
| MacAllister 2015  [13] | RCT | 406 | RIC (early)47.6, 15.1  (late)45.9, 14.2  Control  46.8, 15.1 | RIC 72.4  65.7  Control 61.1 | NR | NR | RIC (early) 6.86, 2.89  Late 7.03, 3.35  Control 7.18, 3.30 | NR |
| Veighy  2019  [14] | RCT | 406 | NR | RIC 72.4  65.7  Control 61.1 | NR | NR | NR | NR |
| Zapata-Chavira 2019[15] | RCT | 29 | RIC 49.9 ± 13.6  Control 56.2 ± 10.8 | RIC 70.5%  Control 66.6% | RIC 29.4%  Control 41.6% | RIC 17.6%  Control 33.3% | NR | NR |

SDC; Table 1: Baseline characteristic for each included study (KIDNEY TRANSPLANT RECIPIENTS) The baseline characteristics of the population include age, gender, diabetes, hypertension, pre-operative glomerular filtration rate (GFR) and serum creatinine.

|  | | | | | | | |
| --- | --- | --- | --- | --- | --- | --- | --- |
| Study name | Study type | Study Population | Age | Males | MELD | AST  IU/L | ALT  IU/L |
| Robertson 2017 [16] | RCT | 40 | RIC  55 (±10)  Control  54 (±9) | RIC 90%  Control 80% | RIC  15 (±5)  Control 13 (±5) | NR | NR |
| Qi 2021 [17] | RCT | 106 | RIC 30 (27, 34.5)  Control  29 (26, 33.5) | RIC  Control | RIC  Control | RIC 18.0 (14.5, 21.5)  Control  17.0 (15.0, 20.0) | RIC 15.0 (11.0, 21.0)  Control  13.0 (11.5, 16.5) |
| Jung 2020  [18] | RCT | 146 | RIC 56 (49.0, 60.0)  Control 56 (51.0, 60.0) | RIC 68.9%  Control 76.4% | RIC 11(8-18)  Control 11 (9-14) | RIC 30 (25-45)  Control 39 (26-54) | RIC 19.0(13-27)  Control  22.5 (16-41) |
|  |  |  |  |  |  |  |  |
|  |  |  |  |  |  |  |  |

SDC; Table 2: Baseline characteristic for each included study (LIVER TRANSPLANT RECIPIENTS). The baseline characteristics of the population include age, gender, Model for End-Stage Liver Disease (MELD) score , aspartate aminotransferase (AST), and alanine transaminase (ALT).

**
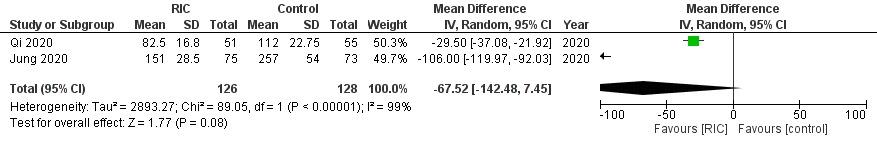
**

SDC; Figure 1: Forest plot of comparison: RIPC vs Control, outcome: ALT at 1 week post-operatively in liver transplant patients

*
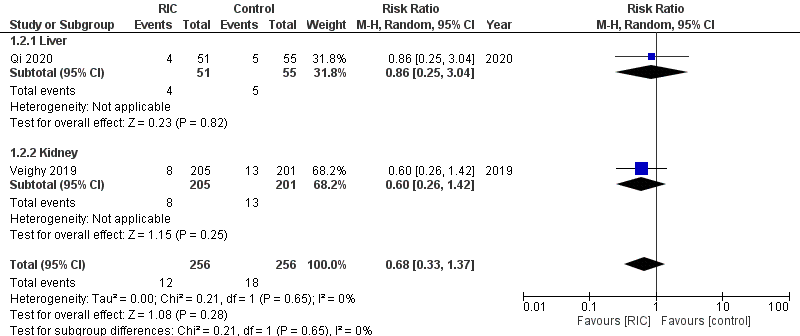
*

SDC; Figure 2: Forest plot of comparison: RIPC vs Control, outcome: Mortality at follow-up in both kidney and liver transplant patients

**
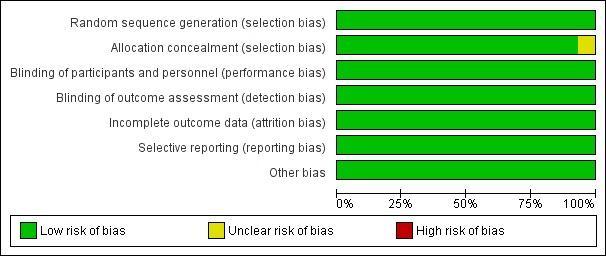
**

**
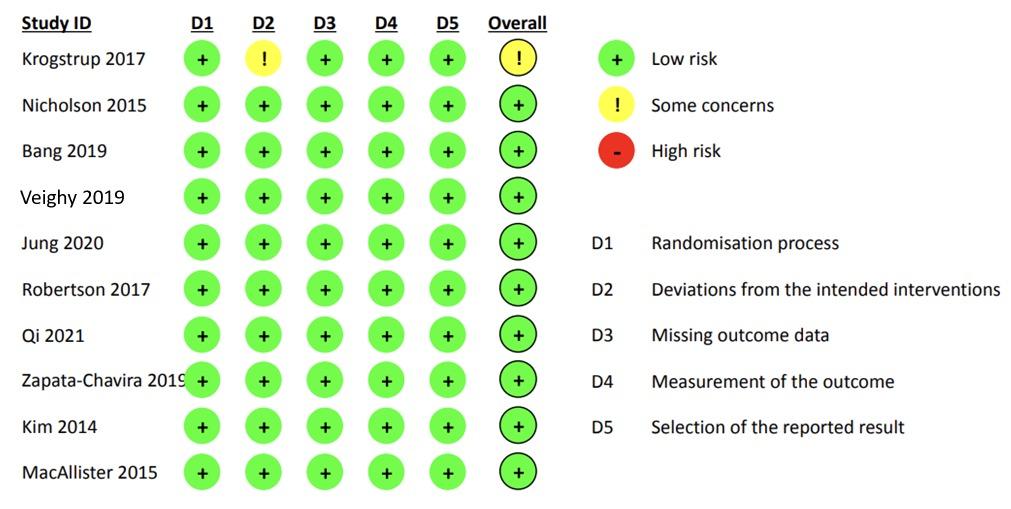
**

SDC; Figure 3: Cochrane risk-of-bias tool for randomized trials (RoB 2.0)
